# Governance power of actors in building safe community environment

**Published:** 2019-08

**Authors:** Leila Doshmangir, Saeid Pour Doulati, Rahim Khodayari-Zarnaq

**Affiliations:** ^*a*^Tabriz Health Services Management Research Center, Iranian Center of Excellence in Health Management, Department of Health Services Management, School of Management and Medical Informatics, Tabriz University of Medical Sciences, Tabriz, Iran.; ^*b*^Social Determinants of Health Research Canter, Health Management and Safety Promotion Research Institute, Tabriz University of Medical Sciences, Tabriz, Iran.; ^*c*^Center for Non-Communicable Diseases Control and Prevention, East Azerbaijan Province Health Center, Tabriz, Iran.

## Abstract

**Background:**

The government power of actors and their influence on changing policies could result in changes in health outcomes as well as health indicators. Health and wellness are more influenced by the places in which people live, learn, work, and play. Building a safe community environment depends on the governance power of actors. Purpose: The aim of this study is to have an overview of the key actors of the safe communities and compare the governance power of them across a sample of designated safe communities.

**Methods:**

The present study is a combination of a qualitative study with a document review method and a comparative review study. Initially, upstream documents, reports on stakeholders’ participation in the creation of safe communities were reviewed. The data were analyzed through content analysis. Then through a comparative study, the organizational structures, key actors, and government power of them were compared in a sample of designated safe communities.

**Results:**

Collaborative and participatory involvement is an essential factor for the creation of safe communities, which has been emphasized by the upstream and international documents. More convergence of the policies and programs across the spectrum of actors (governmental/non-governmental) is needed. The governance power of actors will be flourished by creating a systematic and structured network that will lead to the creation of safe communities.

**Conclusions:**

All actors must participate in the creation of a safe community and use their governance power for this purpose. But the evidence indicates that the most important actors, people, is being ignored. It is imperative that authorities have adequate planning to engage in systematic and continuous community participation by advocating for establishing the not for profit organizations and charities to build and maintain networks for safe communities.

**Keywords:**

Governance power, Safe community, Key actors, Community participation

